# T cell exhaustion in *Giardia intestinalis* infection: A review

**DOI:** 10.1016/j.parepi.2026.e00500

**Published:** 2026-03-27

**Authors:** Williams Walana, Frank Yelevuuro Dasaa, Jennifer Suurbaar

**Affiliations:** a University for Development Studies, School of Medicine, Department of Clinical Microbiology, Tamale, Ghana; b University for Development Studies, School of Medicine, Department of Biochemistry and Molecular Medicine, Tamale, Ghana

**Keywords:** T cell exhaustion, Immune exhaustion, Giardiasis, *Giardia lamblia*, *Giardia intestinalis*, *G. Duodenalis*

## Abstract

**Background:**

Giardiasis, caused by the protozoan parasite *Giardia intestinalis*, is a gastrointestinal infection responsible for approximately 280 million cases of gastroenteritis worldwide each year. While T cells are crucial in the host's defence against *G. intestinalis*, it is unclear which T cell subsets actively help control the infection.

**Objective:**

This review aims to synthesise existing literature on the relationship between T cell exhaustion and giardiasis, examine the T cell subsets affected, identify diagnostic markers that reflect the severity of *Giardia* infection, and explore potential vaccine development.

**Methodology:**

A comprehensive database search from Google Scholar and Medline/PubMed identified eligible studies that reported on T cell exhaustion markers, immune response and giardiasis. Search terms included “Giardia”, “Giardiasis”, “T cells” and “T cell exhaustion”. Articles were reviewed for data on T-cell function, cytokine expression, and immune responses in people with giardiasis.

**Results:**

T cell exhaustion is common in giardiasis, as indicated by the findings. There are higher proliferation rates and upregulation of surface activation markers in CD4 cells in *Giardia* infection. Natural regulatory T cell levels (CD4+, CD25+, and Foxp3+) are significantly increased in *Giardia* infection. Chronic giardiasis and co-morbidity contribute to T-cell exhaustion.

**Conclusion:**

The activation and proliferation of T cells are key to the clearance of *Giardia* infection. Most cytokine profiles are markedly elevated during *Giardia* infection. Cytokine responses vary depending on host immunity and parasite load. Understanding the roles of cytokines in both T-cell and B-cell-mediated immunity could guide treatment protocols and control measures.

## 1 Introduction

The intestinal protozoan *Giardia intestinalis* causes giardiasis. It is also referred to as *G. duodenalis* or *G. lamblia* (Saghaug et al., 2016; Hajare et al., 2022; Escobedo et al., 2014). It is the most common gastrointestinal tract infection globally. It is a binucleated, flagellated anaerobic or microaerophilic unicellular diplomonad that affects humans and animals (Buret et al., 2015).

The cyst is the infective and environmentally resistant form, while the trophozoite is the proliferative form of the parasite that causes pathology in the small intestines. The trophozoites colonise the epithelial cells of the duodenum and the jejunum of the small intestine (Hadi et al., 2022; Mariana and Das, 2016). *G. intestinalis* is a multispecies complex that has been molecularly characterised into eight assemblages, A-H, and assemblages A and B, mainly affecting humans (Buret et al., 2015; Tijani et al., 2023; Klotz and Aebischer, 2015). Assemblages C, D, E, and F have also been reported to affect humans (Tijani et al., 2023).

According to WHO reports (2010), *Giardia* infection is estimated to cause about 28.2 million cases of diarrhoea annually. The prevalence of giardiasis depends on several factors, such as geographic region, socioeconomic status, sanitation, and access to clean water (Dumevi et al., 2025; Anim-baidoo et al., 2016). The prevalence ranges from 2 to 5% in industrialised countries and from 20 to 30% in resource-limited countries (Hajare et al., 2022). In developed countries, this is attributable to urbanisation, international travel, and tourism (Cotton et al., 2015). The parasite is associated with poor sanitation, poverty, poor personal hygiene, and unclean water (Hajare et al., 2022).

The transmission of this protozoan occurs via the oral-fecal route, following direct or indirect contact with infectious stages, including person-to-person, waterborne, and foodborne routes (Buret et al., 2015). It can also be transmitted through oral-anal sex and among homosexuals (Escobedo et al., 2014). Children in daycares, travellers in endemic regions, cystic fibrosis patients, and the immunocompromised are usually at risk of giardiasis (Buret et al., 2015). Humans with common immunodeficiency and impaired IgA are likely to develop chronic giardiasis (Saghaug et al., 2016). Other risk factors that are considered in diagnosing infections include endemicity and exposure to contaminated water and food (Escobedo et al., 2014).

Differences in clinical presentations may be attributed to the host's age, immune and nutritional status, coexisting infections, and the virulence and pathogenicity of *Giardia* strains (Shihab Ahmed et al., 2015). Affected individuals are mostly asymptomatic. However, it presents severe diarrhoea in children, associated with dehydration, abdominal cramps, nausea, weight loss, and malnutrition (Saghaug et al., 2016; Buret et al., 2015). Loss of fluids and electrolytes due to dehydration will lead to electrolyte imbalance and shock, and can be fatal (Shihab Ahmed et al., 2015). Most *Giardia* infections are considered subclinical and self-limiting. However, reinfection has been recorded in endemic areas (Babaei et al., 2016). In children, it is severe when compared to adults, and it is associated with malabsorption, retarded growth and poor care (Hajare et al., 2022). Chronic giardiasis in children can lead to deficits in physical and cognitive development (Gil et al., 2018). The higher morbidity in children is due to poor personal hygiene, consuming unwashed fruits, unhygienic toilet practices, and drinking contaminated water (Hajare et al., 2022).

The presence of trophozoites or cysts in a stool sample is a definitive diagnosis for giardiasis (Escobedo et al., 2014; Hadi et al., 2022). Individuals usually present offensive, bulky, pale, non-bloody, mucoid, or watery stool (Hajare et al., 2022). Enzyme-linked immunosorbent assay and direct fluorescent antigen detection methods are more sensitive than microscopy for diagnosing giardiasis (Dumevi et al., 2025).

The humoral and cell-mediated immune responses contribute to acquired immunity (Shihab Ahmed et al., 2015). White blood cells contribute to the acquired immune response by secreting mucus and enzymes that line the gastrointestinal tract and aid bowel movement. It also regulates gastric pH. The body's immune system employs several defensive mechanisms during parasite invasion (Shakir, 2024). Intestinal epithelial cells secrete antimicrobial peptides that help maintain the integrity of the mucosal lining. Mast cells, phagocytes, and dendritic cells are immune cells that are attracted to the mucosa (Shihab Ahmed et al., 2015; Babaei et al., 2016).

T cells play a significant role in the clearance of *Giardia* infections (Fink et al., 2019), and memory responses may last several years (Saghaug et al., 2016). The response of T cells is crucial in the adaptive immune system for removing infectious agents and cancer cells, playing a vital role in managing and eradicating foreign invaders. Nonetheless, in specific chronic conditions, continuous exposure of T cells to elevated antigen levels may lead to significant T-cell dysfunction (Wherry and Kurachi, 2015). T cell exhaustion occurs when T cells lose function during chronic infection, resulting in diminished effector functions, poor responses to restimulation, and inefficient homeostatic proliferation (Gil et al., 2018; Shakir, 2024). Parasitic load, duration of infection, and the immune system affect T-cell exhaustion (Gil et al., 2018; Di Genova and Tonelli, 2016). Molecular methods are utilised to evaluate the presence of markers associated with T cell exhaustion (Saghaug et al., 2016).

Inhibitory receptors send inhibitory signals that reduce T cell activation; these include PD-1 (programmed cell death protein 1), CTLA-4 (cytotoxic T-lymphocyte-associated protein 4), LAG-3 (lymphocyte activation gene 3), TIM-3 (T cell immunoglobulin and mucin domain protein 3), and TIGIT (T cell immunoreceptor with IgT and ITIM domains). These receptors interact with ligands on target cells, initiating signalling cascades that suppress T cell proliferation, cytotoxic activity, and cytokine production (Wherry and Kurachi, 2015; Catakovic et al., 2017).

Effector molecules mediate immune responses by releasing signalling cytokines: interleukin-2 (IL-2), tumour necrosis factor-alpha (TNF-α), interferon-gamma (IFN-γ), and Granzyme B, a cytolytic protein that facilitates target cell lysis and programmed cell death (Catakovic et al., 2017). Interleukin 17 (IL-17) is a significant T cell cytokine necessary for protective immunity against *Giardia* infection (Fink et al., 2019). Cytokines such as IL-6, tumour necrosis factor-beta, gamma interferon and IL-4 are also released by the host immune cells to control *Giardia* infection (Saghaug et al., 2016)**.** However, the activation of T cells and recognition of *Giardia* parasites by a particular innate cell is unclear (Fink et al., 2019).

It is essential to understand the interactions between the host and the protozoan parasite, the immunological components that contribute to infection prevention, and the antigenic characteristics that elicit host immune responses (Sardinha-Silva et al., 2022). This review, therefore, aimed to synthesise existing literature on the relationship between T cell exhaustion and giardiasis, examine the T cell subsets affected, identify diagnostic markers that reflect the severity of *Giardia* infection, and explore potential vaccine development.

## 2 Methodology

### 2.1 Study design

This review spanned from January 2015 to February 2025, adhering to the PRISMA 2020 guidelines for a comprehensive and transparent approach. It involved a structured framework for identification, screening, eligibility, and inclusion, conducted independently to minimise errors and bias. Based on the understanding of T cell exhaustion in giardiasis, articles were selected from peer-reviewed journals. Data extraction encompassed T cell functionality, inhibitory receptor expression, cytokine profiles, and giardiasis.

### 2.2 Search Strategy

**Fig. 1:**
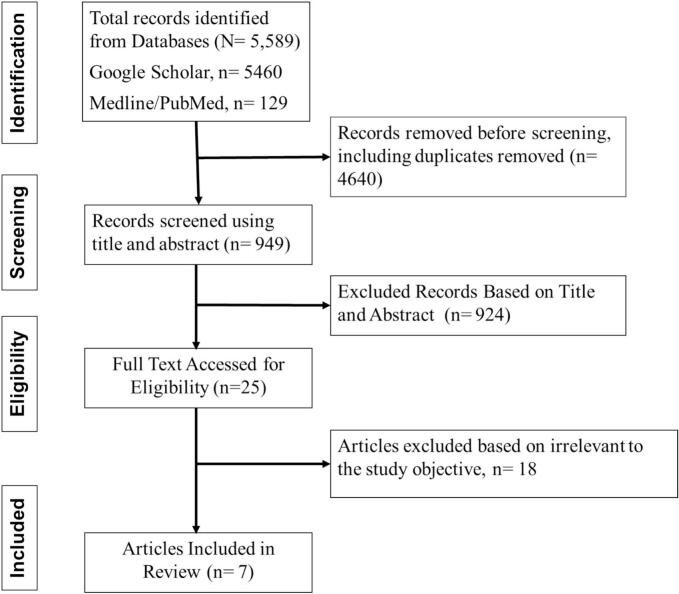
A systematic flowchart of the articles retrieved.

A literature search was conducted using Google Scholar and the Medline/PubMed databases to identify and synthesise findings of previous studies. Applying Medical Subject Headings (MeSH) for Medline/PubMed, keywords were combined using logical operators to develop a comprehensive search strategy. A combination of keywords from the title was used to construct Boolean search strings (“Giardia” OR “*Giardia lamblia*” OR “*Giardia intestinalis*' OR “*G. duodenalis*” OR “Giardiasis”) AND (“T cell exhaustion” OR “T cell”) to search and identify relevant studies. This procedure ensured a comprehensive, methodical approach to understanding the immunological role and T-cell exhaustion in giardiasis. The search process has been summarised in Fig. 1.

### 2.3 Inclusion Criteria

Data and available articles were retrieved based on the following predefined criteria: full articles that are accessible, written, and published in English, experimental and observational published between 2015 and February 2025.

### 2.4 Exclusion criteria

Review articles, letters to the editor, editorials, commentaries, experts' opinions, books and book chapters, brief reports, duplicated studies found in different databases, other articles that are not fully available, and studies using animal models were excluded from this review. These criteria helped to avoid bias in study selection and ensured a thorough search of the data.

## 3 Results

A total of 5589 articles were found in our literature search. These articles were filtered to remove duplicates and irrelevant studies. According to the present inclusion criteria, seven articles were included. The characteristics of selected articles are displayed in Table 1.

**Table 1:** Summary of findings from included articles; T-cells and Exhaustion Markers, GI: *Giardia*-infected participants; HC-Healthy/Low risk control.

#	Reference	Population Characteristics	T-cells and Exhaustion Markers	Practical Implications
1	(Hadi et al., 2022)	*N* = 67, GI = 67, HC = 20	IL-17 and IL-35 levels were higher among infected subjects.	Increased levels of IL-17 and IL-35 indicate the role of interleukins during *Giardia* infection.
2	(Shihab Ahmed et al., 2015)	*N* = 255, GI = 60, HC = 20	Elevated levels of IL-6 and TNF were recorded among the infected subjects. The levels of IL-8 and IL-10 between infected and non-infected were not significant.	IL-6 and TNF-α could be considered immune response markers to giardiasis.
3	(Saghaug et al., 2016)	*N* = 21 GI = 21 HC = 12	Elevated levels of tumour necrosis factor alpha, and IL-17 A-producing CD4 effector memory T cells. The number of IL-17A-producing CD4 effector memory (EM) T cells increased in individuals exposed to *Giardia*.	These findings indicate that IL-17A production is a significant component of the CD4 EM T cell response triggered by symptomatic *Giardia* infection. There may be increased proliferation and upregulation of surface activation markers on CD4 T cells compared with low-risk controls.
4	(Kizilbash et al., 2023)	*N* = 5, GI = 33, HC = 18	There was a significant increase in the levels of CD4+, CD25+, and Foxp3+ in *Giardia*-infected subjects.	Parasitic infection is a risk factor for higher natural regulatory T cells.
5	(de Queiroz et al., 2024)	*N* = 25, GI = 15, HC = 10	Increased regulatory T cells and TGF-β in *Giardia-*infected subjects. Decreased levels of melatonin in IgM-*G. intestinalis-*infected mothers. Increased levels of melatonin in IgG reactive mothers.	*Giardia* infections favour a regulatory environment and are an indicator of symptomatic infections. Parasites may facilitate the progression of a recent infection and disrupt melatonin production.
6	(Shakir, 2024)	*N* = 90, GI = 90	Decreased level of IL-10 in *Giardia*-infected subjects. Both TLR2 and TLR4 levels were high compared to the healthy control group	The low levels of IL-10 could be due to weakening the host's defences against infection.
7	(Babaei et al., 2016)	*N* = 78, GI = 40, HC = 38	An increased level of interferon-gamma in symptomatic subjects and subjects infected with genotype A1 of *G. intestinalis.* Serum IL-10 level was increased in subjects infected with *Giardia* genotype A1. In both asymptomatic subjects and healthy controls, similar interferon levels were detected.	The findings suggest the roles of host and parasite factors in the outcome of enteric infections.

The key findings from the study are summarised in Table 1**.** *Giardia* infection triggers a complex immune response that comprises both pro-inflammatory and regulatory systems. Marked elevations in IL-17 levels were noted in infected individuals, accompanied by an increased quantity of IL-17 A-producing CD4 effector memory (EM) T cells. In the same cohort of patients, IL-35 levels were elevated, with higher levels observed in healthy controls (Hadi et al., 2022). Supporting the rise in IL-17, an increase in IL-17 A-producing CD4 effector memory (EM) T cells in individuals exposed to *Giardia*, along with a higher number of cells simultaneously producing both IL-17 A and TNF-α (Saghaug et al., 2016).

The mean concentrations of IL-6 and TNF-α were higher in *Giardia*-infected individuals than in uninfected ones. However, IL-8 levels did not differ significantly between the two groups (Shihab Ahmed et al., 2015). IL-10 results were inconsistent: one study found no significant difference, another demonstrated a reduction, and those with the AI genotype exhibited higher IL-10 levels.

Individuals infected with *Giardia* exhibited a substantial increase in natural regulatory T cells (CD4+, CD25+, and Foxp3+) compared with healthy controls. CD4+, CD25+, and Foxp3+ levels were significantly increased in *G. intestinalis* infection relative to other parasite illnesses. Individuals with combined illnesses exhibit higher levels than those with single infections (Kizilbash et al., 2023).

In maternal responses, the percentage of regulatory T cells in colostrum was higher in IgM-reactive mothers than in the control group. These mothers also had increased TGF-β levels. Cortisol and IL-10 levels were similar between groups. However, melatonin levels were higher in IgG-seropositive mothers and lower in IgM-reactive mothers. There was no correlation between melatonin, cortisol, and cytokines, although IL-10 and TGF-β were present in colostrum (de Queiroz et al., 2024).

In symptomatic human *Giardia* infection, a significantly higher level of IFN-γ was recorded compared to healthy controls. However, IFN-γ levels among asymptomatic human subjects and controls were similar. Individuals with genotype AI exhibited high IFN-γ concentrations, whereas those with genotype AII had levels comparable to those of healthy controls. IL-5 levels were lower in both infected and control subjects with genotype AII (Babaei et al., 2016). Also, there was an increased concentration of TLR2 and TLR4 in infected participants compared to uninfected subjects (Shakir, 2024).

## 4 Discussions

In giardiasis, T cells play a vital role in pathogen clearance (Saghaug et al., 2016). The immune system has several ways to curb infections, and many of these processes result in the activation of cytokines. However, it is unclear which cytokines actively contribute to the control of *G. intestinalis* and which ones are inactive during infection (Shihab Ahmed et al., 2015). During *Giardia* infection, most of these interleukins are generated in the mucosa-associated lymphoid tissue in response to antigenic stimulation. *G. intestinalis* is an extracellular parasite, and the immune response is stimulated by T helper 2 cells. Cellular immunity is mediated by T helper 2 cells, which orchestrate responses to intracellular pathogens, including bacteria and viruses (Shakir, 2024).

Toll-like receptor 2 (TLR2) is a membrane protein that recognises foreign objects and transmits signals to cells of the immune system. TLR4 is a key component of innate immunity and recognises molecules on the intestinal surface. Infestation with *Giardia* triggers an immunological response by detecting *Giardia*-associated molecular patterns, thereby initiating an immune cell signalling cascade. It activates antigen-presenting cells, leading to the production of pro-inflammatory cytokines (IL-6, TNF-α, IFN-γ) (Liu et al., 2021a), and the recruitment of other cells to fight the infection. It also modulates mechanisms such as A20 expression to balance pathogen clearance and limit tissue damage (Liu et al., 2021b). The increase in TLR2 level indicates its activation by *G. intestinalis.* Both TLR2 and TLR4 expressions in *Giardia*-treated macrophages were higher (Liu et al., 2021a). Understanding the process by which TLR2 is activated during *G. intestinalis* infection is crucial for elucidating host defence mechanisms and potential therapeutic targets. Adaptor proteins, such as TIR-domain-containing adaptor-inducing interferon-β (TRIF) and myeloid differentiation factor 88 (MyD88), engage TLR4 upon activation, initiating downstream signalling pathways. Stimulation of pro-inflammatory cytokines attracts immune cells to the sites of infection and promotes inflammation (Shakir, 2024; Liu et al., 2021a).

The findings indicate that IL-17 and IL-35 were assessed in *Giardia*-infected individuals, revealing higher IL-17 levels across all affected age groups (Hadi et al., 2022). The density of parasites and the host's immunological response significantly affect interleukin concentrations. IL-17-producing immune cells play a crucial role in mucosal defence against infection, inducing cytokines and chemokines that regulate the innate immune response, including neutrophils, dendritic cells, and macrophages (Saghaug et al., 2016; Hadi et al., 2022). Higher levels of IL-35 indicate an anti-inflammatory role of the cytokine, and it downregulates inflammation by inducing regulatory T cells and suppressing T helper 1 and T helper 17 cells (Hadi et al., 2022).

IL-17 A is a polyfunctional cytokine that is involved in regulating mucosal homeostasis, inflammation, and immunity (Dann et al., 2015). Higher levels of IL-17 A recorded in various studies indicate that the cytokine plays a critical role in mucosal defence against *Giardia* (Dann et al., 2015). IL-17 A levels are considerably enhanced during *G. muris* infection in mice, with expression peaking occurring around day 21 post-infection, indicating an active immunological response (Paerewijck et al., 2017). IL-17 A has been reported to play a crucial role in protection after infection or vaccination. Because IL-17 A is a polyfunctional responsive effector memory T cell, those with a strong IL-17 A response may be able to eliminate *Giardia* faster than those with a weak IL-17 A response (Saghaug et al., 2016). IL-17 A has been recorded to be the most strongly induced IL-17 family member after infection (Dann et al., 2015). Several studies have found that animals lacking IL-17 A or its receptor, IL-17RA, are unable to clear *Giardia* infections, emphasising the role of IL-17 A in protective immunity (Paerewijck et al., 2017).

IL-6 is derived from dendritic cells and mast cells (Bartelt and Sartor, 2015), stimulates B-cell differentiation and antibody production, T-cell proliferation, and modulates the IgA response. The increased level is necessary for early control of acute giardiasis (Shihab Ahmed et al., 2015; Paerewijck et al., 2017). An increased level of IL-6 in mast cells upon stimulation with *Giardia* soluble antigen in a dose-dependent manner compared with non-stimulated cells (Muñoz-Cruz et al., 2010). Mast cells play a crucial role in the early immune response to giardiasis. It secretes IL-6, which is essential for managing the infection.

IL-6 and IL-17 control parasites; they may also cause microbial translocation, increased permeability, and disruption of the intestinal barrier. However, immune-mediated inflammation may contribute to crypt hyperplasia, villus atrophy, mucosal damage, and nutrient malabsorption, all of which hinder the growth and development of affected populations (Fink and Singer, 2017).

IL-10 promotes the synthesis of TNF-α and IFN-γ by natural killer cells (NK), enhances phagocytic activities, stimulates the proliferation of activated B cells, and helps their differentiation into cells that secrete antibodies (Escobedo et al., 2014; Sardinha-Silva et al., 2022). It regulates the immune response, protecting the intestinal epithelial barrier and minimising host cell death. In addition, inflammation causes tissue damage (Shakir, 2024).

The increased expression of the immune-regulatory cytokine IL-10 is likely to help avoid additional damage (Paerewijck et al., 2017). However, data on IL-10 levels in *Giardia* infections are conflicting: one study reported low concentrations of both IL-8 and IL-10 (Shihab Ahmed et al., 2015), whereas other studies have reported increased IL-10 production in humans infected with *G. intestinalis* and have attributed this to its anti-inflammatory role (Khalaf et al., 2021). Variations in IL-10 concentrations may be attributable to the study population, the genetic diversity of parasite strains, the severity of infection at the time of sample collection, and the specific immune responses elicited by different parasite genotypes (Babaei et al., 2016). The lower concentration suggests a minor role for T helper 2 cytokines in the immune response to giardiasis. Th2 promotes cellular immunity. (Shakir, 2024). Notably, a reduction in IL-10 suggests a weakened regulatory mechanism, resulting in insufficient suppression of inflammatory responses. As a result, giardiasis symptoms may be exacerbated, with heightened inflammation and tissue damage (Shakir, 2024).

The review found that an increased TNF-α (Saghaug et al., 2016; Shihab Ahmed et al., 2015), a mediator of inflammatory response (neutrophil activation) and cell survival, also plays a vital role in parasitic diseases. TNF-α, a critical mediator of inflammation, may help eliminate *Giardia* by promoting cell survival, enhancing immune cell function, and amplifying the overall immune response. Excessive TNF-α production can cause tissue inflammation, suggesting a role in both protective immunity and inflammation associated with giardiasis (Saghaug et al., 2016).

In symptomatic individuals, significantly higher levels of IFN-γ were observed compared to asymptomatic carriers and healthy controls. This indicates a robust Th1 immune response during the acute phase of the disease. Additionally, individuals infected with the AI genotype exhibited markedly higher IFN-γ levels, suggesting that this genotype elicits stronger immunological activation, which may be associated with more severe clinical symptoms. In contrast, those infected with the AII genotype presented comparable levels of IFN-γ to healthy controls. This reflects a less severe immunological response to giardiasis (Babaei et al., 2016).

The variance in IFN-γ levels dependent on genotype AI vs. AII highlights how parasite heterogeneity affects illness severity. Genotype AI appears to trigger a more robust Th1 response, which may explain the more severe clinical symptoms compared to the less immunogenic AII genotype. In symptomatic cases, increased IFN-γ levels indicate a multifaceted role: the cytokine regulates protective immunity while also promoting immunopathology, depending on the systemic context. Generally, genotype-specific changes in IFN-γ production demonstrate that parasite genetic diversity influences host immune responses and regulates the severity of giardiasis (Babaei et al., 2016).

During tissue injury and inflammation, IL-8 is a major chemokine responsible for neutrophil recruitment; therefore, it has little or no influence on immunity to *Giardia* infection (Shihab Ahmed et al., 2015). In *Giardia* infection, the low concentration of IL-8 may be attributed to the parasite's ability to evade host immune responses. The inhibition of IL-8 synthesis suggests a strategic shift by the parasite to facilitate its colonisation in the host's intestinal environment (Faria et al., 2020).

Inhibitory cytokines, such as TGF-β, are produced and released by CD4 + CD25 + FOXP3+ regulatory T cells to suppress the cellular immune response and halt the recruitment of inflammatory cells (de Queiroz et al., 2024). While the host induces this immunoregulatory response to limit tissue damage, it also results in continuous antigen exposure and engagement of inhibitory receptors, which may ultimately drive T-cell exhaustion. In this state, T cells lose their vital ability to proliferate and secrete effector cytokines. Interestingly, *Giardia* induces a higher proportion of these regulatory cells than other parasitic infections, likely due to its minimal inflammatory response. This regulatory dominance is further amplified in mixed infections and has been observed notably in children infected with *G. intestinalis* (Kizilbash et al., 2023).

The persistently high levels of IFN-γ, TNF-α, and IL-17 A indicate that numerous individuals sustain a vigorous, albeit possibly regulated, effector response. The data strongly suggest an immune regulatory or modulatory profile rather than a purely exhausted one. The notable and persistent rise in natural regulatory T cells (CD4 + CD25 + FOXP3+) and the presence of inhibitory cytokines such as TGF-β indicate that the immune system is actively reducing its response to mitigate intestinal tissue damage, rather than merely becoming exhausted.

Due to antigenic variation and strain diversity, vaccine development for giardiasis has been a significant challenge. There is currently no human vaccine against giardiasis (Fink and Singer, 2017; Khalaf et al., 2021). However, a less efficacious *Giardia* VAX has been approved for veterinary purposes. Various immunogens of *G. intestinalis*, including variant-specific surface antigens (VSPs), giardins, cyst wall protein 2, excretory-secretory products (ESPs), and immunoglobulin-binding protein (BIP), have been exploited as human vaccine candidates (Khalaf et al., 2021; Faria et al., 2020). Further studies are needed to investigate the mechanisms of exhaustion in giardiasis and to optimise these immune responses for targeted vaccines to curb the infection. Therapeutic interventions targeting T cell exhaustion hold promise for improving the treatment of chronic giardiasis (Gigley et al., 2012). However, studies are needed to evaluate their safety and efficacy. Additionally, the genotype-specific immune responses (AI vs. AII) underscore the need for diagnostic markers that account for parasite genetic diversity.

## 5 Conclusion

This review investigated the relationship between T cell exhaustion and *Giardia* infection. The review indicates that different T cell subsets are vital for the clearance of giardiasis. The infection initiates a Th2 response, and activation of both TLR2 and RLR4 occurs. Cytokine responses differed with host immunity and parasite load.

Interleukin-17 (IL-17) levels were consistently found to be elevated among all cases of giardiasis. IL-6 and TNF-α were also elevated in giardiasis. While IL-8 and IL-10 levels were mainly low, some studies recorded increased IL-10 levels. A significant finding across most studies was the elevated levels of CD4 + CD25 + Foxp3+ regulatory T cells, particularly among children. While these points collectively indicate a complex immune response during *Giardia* infection, the precise mechanism of T cell exhaustion remains unclear. Based on the finding, the memory CD4+ T-cell response to giardiasis could be enhanced for therapy and vaccine development in individuals in endemic regions. Given the status of giardiasis as a neglected disease, it is essential to involve public health organisations in implementing strategies for its prevention and control.

## CRediT authorship contribution statement

**Williams Walana:** Writing – review & editing, Writing – original draft, Data curation, Conceptualization. **Frank Yelevuuro Dasaa:** Writing – review & editing, Writing – original draft, Data curation. **Jennifer Suurbaar:** Writing – review & editing, Writing – original draft, Data curation, Conceptualization.

## Author contributions

WW, FD, and JS conceived the study. They reviewed and selected the articles for this review. All the authors made specific contributions to the development of this manuscript and approved the final version.

## Funding

The study received no external funding.

## Declaration of competing interest

The authors declare that they have no known competing financial interests or personal relationships that could have appeared to influence the work reported in this paper.
